# Prognostic biomarker MCP-4 triggers epithelial-mesenchymal transition *via* the p38 MAPK pathway in ovarian cancer

**DOI:** 10.3389/fonc.2022.1034737

**Published:** 2022-12-01

**Authors:** Siting Li, Yuexin Hu, Ouxuan Liu, Xiao Li, Bei Lin

**Affiliations:** ^1^ Department of Obstetrics and Gynecology, Shengjing Hospital of China Medical University, Shenyang, China; ^2^ Key Laboratory of Maternal-Fetal Medicine of Liaoning Province, Key Laboratory of Obstetrics and Gynecology of Higher Education of Liaoning Province, Shenyang, China

**Keywords:** p38 MAPK signaling pathway, apoptosis, EMT, ovarian cancer, MCP-4

## Abstract

**Background:**

Monocyte chemoattractant protein-4 (MCP-4/CCL13) is a proinflammatory factor that is overexpressed in various malignant tumors and may play an important role in tumor progression and metastasis. However, its role and mechanism of action in ovarian cancer remains unknown.

**Methods:**

Immunohistochemistry (IHC) was performed to detect the expression of MCP-4 in ovarian cancer tissues, and the effect of MCP-4 on patient survival and prognosis was analyzed. Overexpression and suppression of MCP-4 in ovarian cancer cell lines were then established, and their effects on cell invasion, migration, and apoptosis were studied. ES-2 cell lines were employed to establish a peritoneal dissemination model in nude mice. Western blotting was performed to detect the expression of epithelial mesenchymal transition (EMT) markers and the p38 mitogen-activated protein kinase (p38 MAPK) signaling pathway.

**Results:**

MCP-4 was highly expressed in ovarian cancer tissues and its expression level was related to the prognosis of patients with ovarian cancer. MCP-4 overexpression promoted the migration and invasion of ovarian cancer cells but inhibited apoptosis. MCP-4 overexpression increased the expression of MMP-2, MMP-9, N-cadherin, vimentin and Bcl2/Bax and decreased the expression of E-cadherin. MCP-4 overexpression increased the phosphorylation of the p38 MAPK pathway. The inhibition of MCP-4 expression indicated an opposite trend. *In vivo* experiments have also confirmed that MCP-4 overexpression can promote metastasis of ovarian cancer.

**Conclusion:**

MCP-4 promotes ovarian cancer progression through the p38 MAPK signaling pathway, and may be a potential biomarker and therapeutic target for ovarian cancer.

## Introduction

Ovarian cancer has the highest death rate among gynecological malignancies, with a five-year survival rate of less than 50% (1). Due to the lack of typical clinical symptoms in the early stages of ovarian cancer, patients are often diagnosed at an advanced stage, when the tumor is difficult to remove completely (2). Despite urgent clinical needs and substantial research efforts, the mainstream treatment of ovarian cancer remains cytoreductive surgery and adjuvant chemotherapy, without much recent progress (3). This further contributes to the fact that ovarian cancer remains a challenge. Therefore, pathogenesis identification of ovarian cancer and new treatment strategies are crucial for improving the survival rate of patients with ovarian cancer.

The human *MCP-4/CCL13* gene is found in the q11.2 segment of chromosome 17, within the chemotactic chemokine (CC) gene cluster (17q11-32), including three exons, two introns, and regulatory sequences near the 5’ upstream region. MCP-4 can bind to chemokine receptors CCR1, CCR2, CCR3, and CCR5 and has chemotactic effects on multiple immune cells, including monocytes, eosinophils, basophils, macrophages, T cells, B cells, and dendritic cells (DCs) (4, 5). In addition, MCP-4 is involved in processes such as eosinophil degranulation, histamine release from basophils, expression of adhesion molecules, and cytokine secretion from epithelial, endothelial, and muscle cells (6). Previous studies on MCP-4 have mainly focused on inflammatory diseases, however, more recent studies have reported that the expression of MCP-4 is increased in colorectal (7) and gastric cancers (8), which are associated with the development of malignant tumors. The expression of MCP-4 in ovarian cancer and its effect on malignant biological behavior have not yet been reported.

In this study, the expression of MCP-4 in ovarian tissue was detected by immunohistochemistry, and its relationship with clinicopathological parameters was evaluated. Additionally, the prognoses of patients with ovarian cancer were analyzed. *In vivo* and *in vitro* models of MCP-4 overexpression and inhibition were established. The role of MCP-4 in the EMT process in ovarian cancer was explored as well as the mechanism of MCP-4 in ovarian cancer occurrence and development. These findings suggest that MCP-4 can potentially be used as an indicator of ovarian cancer, which can be used for diagnosis and prognosis. Finally, these findings provide new research directions for targeted therapy regimens against ovarian cancer.

## Materials and methods

### Specimen source and clinical data

All 147 paraffin-embedded ovarian tissue samples were obtained from surgical inpatients at the Shengjing Hospital, China Medical University, between 2008 and 2019. The study was approved by the Ethics Committee of the Shengjing Hospital, China Medical University, and all patients provided informed and signed consent. The ovarian malignancy specimens were primary epithelial ovarian tumors, and the patients had not received radiotherapy, chemotherapy, or hormone therapy before surgery. The clinicopathological data of all cases were complete. All tissue sections were diagnosed by experienced pathologists at Shengjing Hospital, China Medical University. The total tissue sections consisted of 9 cases of normal ovarian tissue, 12 cases of benign tumors, 21 cases of ovarian epithelial borderline tumors, and 105 cases of ovarian epithelial malignant tumors. The ages of all patients ranged from 19 to 79 years, with an average of 53 years. All the patients ranged in age from 19 to 79 years, with an average age of 52. The mean ages of patients with normal ovaries, benign tumors, borderline tumors, and malignant tumors were 53 years (34 - 76 years), 47 years (35 - 61 years), 47 years (24 - 79 years) and 55 years (19 - 78 years old), respectively. There were no significant differences between the groups (*P* > 0.05). According to the pathological type, a total of 105 malignant ovarian epithelial tumors were divided into 71 cases of serous carcinoma, 10 cases of mucinous carcinoma, 13 cases of endometrioid carcinoma, and 11 cases of clear cell carcinoma. According to the degree of tumor cell differentiation, the total samples were divided into 54 cases of poorly differentiated carcinoma, 24 cases of moderately differentiated carcinoma, and 28 cases of well-differentiated carcinoma. According to the International Federation of Gynecology and Obstetrics (FIGO, 2014), 28 cases were classified as stage I, 10 cases as stage II, 62 cases as stage III, and six cases as stage IV. According to the absence of lymph node metastasis, 31 patients with lymph node metastasis, 67 without lymph node metastasis, and 8 without lymph node dissection were observed.

### Immunohistochemistry

Paraffin-embedded ovarian tissues were cut into 5 µm serial sections. An immunohistochemical ultrasensitive TM SP (mouse/rabbit) kit (Cat# KIT-9720, MaiXin, China) was used to detect the expression of MCP-4. The rabbit MCP-4 polyclonal antibody was diluted 1:200 (Cat# 820372, ZEN BIO, China). Other reagents and instruments were provided by the Central Laboratory of the Shengjing Hospital, China Medical University. Staining was performed according to the manufacturer instructions on the SP kit. Positive reactions were defined by the presence of brownish-yellow granular deposits in the cell membrane and cytoplasm. A comprehensive score was calculated based on the intensity of staining and the proportion of stained cells. The samples were divided into no staining (0 points), light yellow (1 point), brown-yellow (2 points), and brown (3 points) according to the intensity of staining. The percentage of colored cells was divided into <5% (0 points), 5%–25% (1 point), 26%–50% (2 points), 51%–75% (3 points), and 76%–100% (4 points). The two items were multiplied by the final scores: 0–2 points (−), 3–4 points (+), 5–8 points (++), and 9–12 points (++++). Based on these scores, 0–4 points were defined as the low-expression group and 5–12 points as the high-expression group. Each sample was scored independently by two observers, with a third observer when disagreement arose.

### Cell culture

Ovarian cancer cell lines (Caov3, ES-2) and human ovarian surface epithelial cell line (HOSEpiC) were purchased from the Cell Bank of the Chinese Academy of Sciences (Shanghai Institute of Biochemistry and Cell Biology, Shanghai, China). Caov3 is a cell line with epithelial morphology isolated in 1976 from the ovary of a 54-year-old, White, female ovarian adenocarcinoma patient. ES-2 is a cell line exhibiting fibroblast-like morphology that was isolated from the ovary of a 47-year-old, Black, female clear cell carcinoma patient. The Caov3 and HOSEpiC cells were cultured in RPMI1640 medium containing 10% fetal bovine serum (FBS). The ES-2 cells were cultured in McCoy’s5A medium containing 10% FBS. Cells were cultured at 37°C, 5% CO2, and saturated humidity.

### Cell transfection and establishment of stably transfected cell lines

The Caov3 and ES-2 cells in the logarithmic growth phase were inoculated into six-well plates a day before transfection. MCP-4 siRNA and negative control siRNA (GenePharma, China) were transfected into cells using the liposome method (Lipo3000, Cat# L3000015, GIBCO, Invitrogen, USA). The sequence of MCP-4 siRNA is: sense: 5’- GAAAGUCUCUGCAGUGCUUTT-3’, antisense: 5’- AAGCACUGCAGAGACUUUCTT -3’. The sequence of its negative control is: sense: 5’-UUCUCCGAACGUGUCACGUTT-3’, antisense: 5’-ACGUGACACGUUCGGAGAA TT-3’. Following 48h transfection, cells were collected for real-time quantitative polymerase chain reaction (RT-qPCR), western blotting, and biological assays. Caov3 and ES-2 cells were transfected with lentivirus-mediated vector of MCP-4 (Lot# LV61110906, HANBIO, China) or negative control (Lot# LV61110905, HANBIO, China) to establish MCP-4 overexpression or negative control cell lines. The procedure of transfections was conducted according to the manufacturer’s instructions. The volume of lentivirus to be transfected was calculated according to the infection values of different cells, the number of cells at the time of transfection and the lentivirus titer. 2 μg of polybrene (2mg/ml, Lot# 20211015, Hanbio, China) was added to the culture medium to improve transfection efficiency. After transfection for 48h, stable transfected cells were further screened by puromycin (2 μg/ml, Lot# 20211011, Hanbio, China).

### RT-PCR

Total cellular RNA was extracted using TRIzol (Cat# 9109, Takara Bio, Inc., Shiga, Japan), and the purity and concentration of RNA were determined using UV spectrophotometry. A one-step TB Green PrimeScript RT-PCR kit (Cat# 066A Takara Bio, Inc., Shiga, Japan) was used to reverse transcribe RNA into complementary DNA (cDNA), which was then used as a template for DNA amplification. Reverse transcription conditions were as follows: reverse transcription at 42°C for 5 min, heating at 95°C for 10 s. Amplification conditions: denaturation at 95°C for 5 s and 60°C for 30 s, for a total of 40 cycles. MCP-4 primer sequence: Forward: 5’-GCACTCAACGTCCCATCTAC-3’, Reverse: 5’-TTCTCCTTTGGGTCAGCACA-3’. GAPDH primer sequence: Forward: 5 ‘ -ACAACTTTGGTATCGTGGAAGG-3 ‘, Reverse: 5’ -GCCATCACGCCACAGTTTC-3’. The PCR was performed using a 7500 Fast Real-Time PCR system. Data were analyzed using the 2-ΔΔCT method.

### Western blotting

RIPA cell lysis buffer was used to lyse cells at 4°C for 30 min, followed by centrifugation at 12000× g, 4°C for 30 min. The supernatant was collected, and the protein concentration was determined by the BCA method. The 5× loading buffer (Cat# cw0027S, CWVBIO, China) was added to each sample and denatured at 100°C for 10 min. Protein samples were separated by 10% sodium dodecyl sulfate-polyacrylamide gel electrophoresis (SDS-PAGE) and transferred to polyvinylidene difluoride (PVDF) membranes (Cat# IPVH00010, Millipore, USA). Membranes were blocked with 5% nonfat milk for 2 h and incubated with primary antibodies overnight at 4°C. The primary antibodies we used were as follows: MCP-4 (1:1000, Cat# DF9911, Affinity, USA), BCL2 (1:2000, Cat# 12789-1-AP, Proteintech, China), BAX (1:1000, Cat# AF0120, Affinity, China), MMP2 (1:1000, Cat# 10373-2-AP, Proteintech, China), MMP9 (1:500, Cat# 10375-2-AP, Proteintech, China), E-cadherin (1:2000, Cat# 20874-1-AP, Proteintech, China), N-cadherin (1:2000, Cat# 22018-1-AP, Proteintech, China), vimentin (1:2000, Cat# 10366-1-AP, Proteintech, China), p38 MAPK (1:1000, Cat# 8690, Cell Signaling Technology, USA), p-p38 MAPK (1:1000, Cat# 4511, Cell Signaling Technology, USA), and GAPDH (1:2000, Cat# TA-08, ZSGB-BIO, China). The membranes were washed three times with tris-buffered saline tween (TBST) the next day, followed by incubation with a horseradish peroxidase-labeled secondary antibody (1:2000, ZSGB-BIO, China) for 2 h. After washing three times with TBST, the proteins were detected with western blot chemiluminescence horseradish peroxidase (HRP) substrate (Cat# WBKLS0500, Millipore, Billerica, MA, USA) to detect protein expression, and an Amersham Imager 680 (AI680) ultrasensitive luminometer was used for imaging.

### Invasion test

Invasion ability was assessed by Transwell assay in a 24-well culture plate. Matrigel (Cat# 356234, BD Biosciences, USA) and serum-free medium were mixed at a ratio of 1:7.5 and 70 µl was added to the upper chamber of each transwell chamber (Cat# 3422, Corning, USA) and incubated for 4 h at 37°C. A total of 200 µl of serum-free cell suspension (2×10^5^/mL) was added to the upper chamber and 500 µl of RPMI1640 or McCoy’s5A medium containing 20% FBS was added to the lower chamber. After incubation at 37°C and 5% CO2 for 48 h, the cells were fixed with 4% paraformaldehyde for 30 min. The cells were then washed with PBS and stained with 0.1% crystal violet for 30 min. Cells in the upper chamber were gently wiped with a cotton swab. The stained cells were observed and counted under a microscope. At least three random fields were taken photographs for each chamber.

### Wound healing test

A marker was used to mark the back of the 6-well plate every 0.5-1cm, with at least 3 horizontal lines for each hole. Single-cell suspensions were prepared from cells in the logarithmic growth phase and seeded in 6-well plates. When cell fusion reached 90%, the plate was gently scraped with a 200 µl pipette tip. The scratch lines should be perpendicular to the horizontal lines. The cells were washed twice with PBS and cultured in serum-free medium. The wound-healing ability of the cells was observed and photographed under a microscope at 0 and 24 h.

### Apoptosis detection using flow cytometry

The Annexin-V-APC/7AAD (Cat# KGA1025, KeyGEN Biotech, China) double staining method was used to detect apoptosis in ovarian cancer cell lines overexpressing MCP-4, and the Annexin V-FITC/PI (Cat# KGA107, KeyGEN Biotech, China) double staining method was used to detect apoptosis when MCP-4 expression was inhibited. Each group contained a blank control and two types of staining. A blank and single staining control for both dyes were set up for each group. The cells were digested with ethylenediaminetetraacetic acid (EDTA)-free trypsin to obtain a single-cell suspension. Centrifugation (2000 rpm) was performed for 5 min, and the supernatant was removed. A total of 500 μL of 1× Annexin V binding buffer (1×10^6^ cell number/mL) was added to resuspend the cells, and 5 μL Annexin V-APC binding mixture (Annexin V-FITC binding mixture) and 5 μL 7AAD dye (PI dye) were added. After incubation for 15 min in the dark, cell apoptosis was detected using BD FACSDiva software (Cat# 352054, BD Biosciences, USA).

### Nude mouse xenograft model

Twelve 4-week-old female BALB/cA-nu nude mice, purchased from Beijing Huafukang Biosciences (Beijing, China), were maintained in specific pathogen-free conditions. Control vector/MCP-4-overexpressed ES-2 cells (5 × 106) cells were suspended in 150 μL of PBS and injected subcutaneously into the left flank of mice (n = 6). The changes of body weight and abdominal girth were recorded every 4 days. All the mice were sacrificed after 25 days. The animal study was approved by the Institutional Animal Research Committee of China Medical University.

### Bioinformatics analysis

RNA-seq data in TPM (transcripts per million reads) format from The Cancer Genome Atlas (TCGA) database (https://portal.gdc.cancer.gov/) and the Genotype-Tissue Expression (GTEx) database (https://gtexportal.org/) were downloaded. 379 ovarian cancer samples were from TCGA, and 88 normal ovarian tissues samples from the GTEx. Data standardization and log2 transformation were performed for expression comparison between the samples. R software (version 3.6.3) was operated for statistical analysis and visualization. Two datasets (GSE38666 (9) and GSE18520 (10)) were downloaded from the Gene Expression Omnibus (GEO) database (https://ncbi.nlm.nih.gov/geo/). The GSE18520 dataset included 10 normal ovarian epithelial tissues and 53 cases of ovarian epithelial high-grade serous tumors, whereas the GSE38666 dataset included 12 normal ovarian epithelial tissues and 18 ovarian epithelial malignant tumors. These datasets were processed and calibrated, and MCP-4 expression data were extracted using R software (version 3.6.3). The ggplot2 package (version 3.3.3) was used to draw differential expression box plots. GSEA version 4.1.0 software (http://software.broadinstitute.org/gsea/index.jsp) was operated for functional enrichment analysis of the Kyoto Encyclopedia of Genes and Genomes (KEGG) using the data from the TCGA database. The parameters of gene set parameters and run enrichment tests were set as follows. The “c2.cp.kegg.v7.4.symbols.gmt” was chosen as the gene sets database. The permutations value was set to 1000 for computing normalized enrichment score (NES). Signaling pathways with a false discovery rate (FDR) q-value < 0.05 was recognized with significant enrichment. Normalized *P*-value < 0.05 was used to select the enriched signaling pathways when q-value > 0.05.

### Statistical analysis

Data were analyzed using SPSS (version 23.0, IBM Corporation, USA), and graphs were constructed using GraphPad Prism software (version 8.0). All data are presented as mean ± standard deviation. Differences between two groups were compared using the chi-square test and t-test, and differences between more than two groups were compared using analysis of variance. Kaplan–Meier and log-rank tests were used for survival analysis. Univariate and multifactorial Cox regression models were used to analyze the risk factors affecting prognosis. Two-sided *P* < 0.05 was considered statistically significant (*, *P* < 0.05; **, *P* < 0.01; ***, *P* < 0.001).

## Results

### Expression and clinical significance of MCP-4 in ovarian tissues

**Figure 1:**
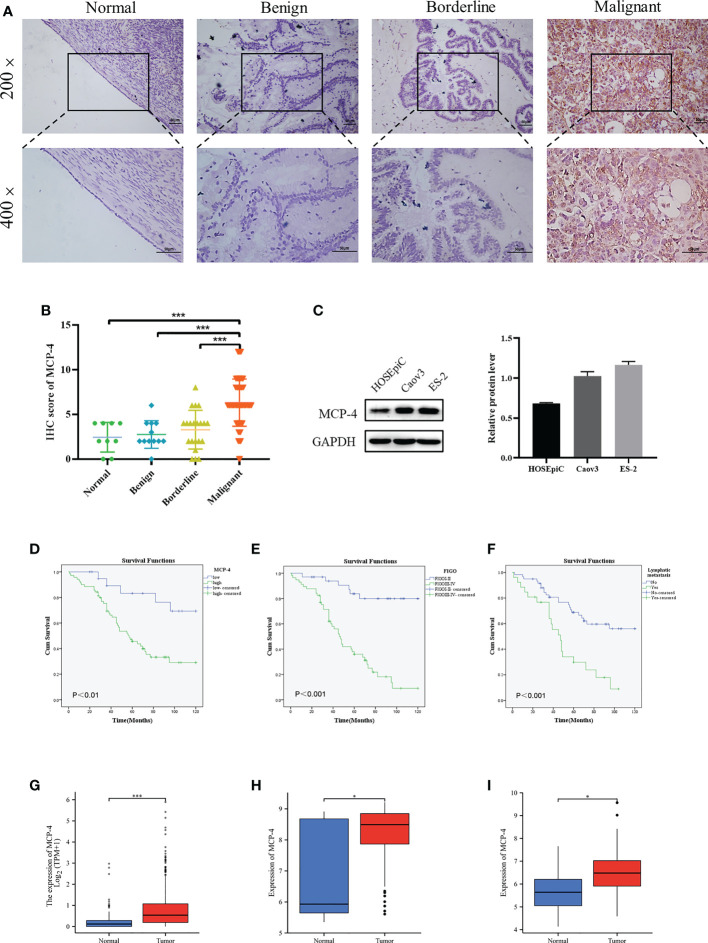
High MCP-4 expression in patients with ovarian cancer associated with poor prognosis. **(A)** MCP-4 expression in ovarian tissue samples (×200 and ×400, scale bar = 50 µm): ovarian normal tissue (n = 9), ovarian benign tumor (n = 12), ovarian borderline tumor (n = 21), ovarian malignant tumor (n = 105). **(B)** Immunostaining scores of MCP-4 in normal ovarian tissues, benign, borderline, and malignant tumors. **(C)** Representative images and quantitation of the western blotting showed that the protein expression of MCP-4 in the normal ovary epithelial cells (HOSEpiC) and ovarian cancer cell lines (Caov3 and ES-2) (n = 3). GAPDH was used as an internal control. **(D–F)** Overall survival analysis according to MCP-4 expression, FIGO stage and lymphnode metastasis. Box plots show MCP-4 mRNA expression in the normal tissues (blue plot) and ovarian tumor (red plot) from TCGA database **(G)**, GSE18520 datasets **(H)**, and GSE38666 datasets **(I)**. Data are presented as mean ± SD. *P < 0.05; ***P < 0.001.

Immunohistochemical results showed that MCP-4 was mainly expressed in the cell membrane and cytoplasm (**Figure 1A**). The positive and high expression rates of MCP-4 in the ovarian cancer group (91.43% (96/105) and 75.24% (26/105)) were significantly higher than those in the borderline group (57.14% (12/21) and 19.05% (4/21)) (both P < 0.05), benign group (41.67% (5/12) and 8.33% (1/12)) (both P < 0.05), and normal ovarian tissues (44.44% (4/9) and 0.00% (0/9)) (both *P* < 0.05) (**Table 1**). In ovarian borderline tumor tissues, the positive and high expression rates of MCP-4 were higher (57.14% (12/21) and 19.05% (4/21), respectively) than in the benign group (41.67% (5/12) and 8.33% (1/12), respectively) (both *P* > 0.05) and the normal group (44.44% (4/9) and 0.00% (0/9), respectively) (both *P* > 0.05) (**Table 1**). The IHC scores for MCP-4 expression are shown in **Figure 1B**. Analysis of normal ovarian tissue data downloaded from the GTEx database and ovarian cancer datasets downloaded from TCGA database showed that the expression of MCP-4 was significantly higher in ovarian epithelial serous cystadenocarcinoma than in normal ovarian tissues (*P* < 0.05) (**Figure 1G**). In addition, the downloaded datasets GSE18520(**Figure 1H**) and GSE38666(**Figure 1I**) from GEO also showed the same results (both *P* < 0.05).

**Table 1:** Expression of MCP-4 in different types of ovarian tissue.

Group	n		Low				High				Positive	High positive
			(-)		(+)		(++)		(+++)		rate (%)	rate (%)
Normal	9		5		4		0		0		44.44	0.00
Benign	12		7		4		1		0		41.67	8.33
Borderline	21		9		8		4		0		57.14^a,b^	19.05^c,d^
Malignant	105		9		17		58		21		91.43^e,f^	75.24^g,h^

^a^Normal vs. Borderline (*P* = 0.002); ^b^Benign vs. Borderline (*P* = 0.061); ^c^Normal vs. Borderline (*P* = 0.060); ^d^Benign vs. Borderline (*P* = 0.031); ^e^Normal vs. Malignant (*P* < 0.001); ^f^Benign vs. Malignant (*P* = 0.001); ^g^Normal vs. Malignant (*P* < 0.001); ^h^Benign vs. Malignant (*P* = 0.001).

According to the IHC results, 105 ovarian cancer samples were divided into the MCP-4 high expression group (+++/++) and MCP-4 low expression group (+/-). The relationship between MCP-4 expression and clinicopathological parameters is shown in **Table 2**. High expression of MCP-4 was significantly correlated with the late International Federation of Gynecology and Obstetrics (FIGO) stage (*P* < 0.05). The high expression rate of MCP-4 in the late FIGO stage (stage III–IV) was 83.08%, which was significantly higher than that in the early FIGO stage (stage I–II) (62.50%). However, the expression of MCP-4 was not correlated with age, histological grade, lymph node metastasis, or pathological type (*P* > 0.05). A total of 105 ovarian cancer patients were followed up until April 1, 2021. Kaplan–Meier survival analysis showed that the overall survival of ovarian cancer patients with high MCP-4 expression was significantly shorter than that of patients with low MCP-4 expression (*P* < 0.05) (**Figure 1D**). FIGO stage (P < 0.05) (**Figure 1E**) and lymph node metastasis (*P* < 0.05) (**Figure 1F**) were significantly correlated with the overall survival.

**Table 2:** Relationship between MCP-4 expression and clinicopathological parameters of ovarian epithelial malignant tumors.

Items	n		Low					High				High positive	*P-*value
			(-)		(+)			(++)		(+++)		rate (%)	
FIGO stage													<0.05
I-II	40		0		15			19		6		62.50	
III-IV	65		0		11			39		15		83.08	
Differentiation													>0.05
Well-Moderate	51		0		15			31		5		70.59	
Poorly	54		0		11			27		16		79.63	
Lymph node metastasis													>0.05
Yes	30		0		9			14		7		70.00	
No	67		0		17			39		11		74.63	
Unknown	8		0		0			5		3		100.00	
Pathological subtype													>0.05
Serous	71		0		18			40		13		74.65	
Mucinous	10		0		4			6		0		60.00	
Endometrioid	13		0		3			8		2		76.92	
Clear cell carcinoma	11		0		1			4		6		90.91	

**Figure 2:**
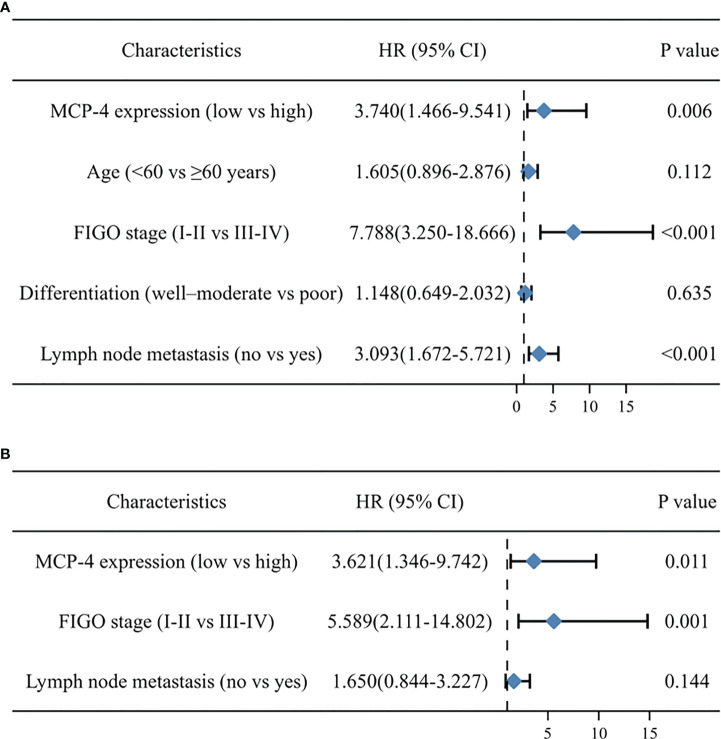
Forest map based on univariate **(A)** and multivariate **(B)** cox regression analyses.

Cox regression analysis of the relationship between different clinicopathological parameters and the prognosis of patients with ovarian cancer univariate Cox regression analysis showed that high MCP-4 expression, advanced FIGO stage, and lymph node metastasis were risk factors affecting the prognosis of patients with ovarian cancer (all *P* < 0.05) (**Table 3**). Multivariate Cox regression analysis showed that high MCP-4 expression and advanced FIGO stage were independent risk factors affecting patient prognosis (all *P* < 0.05) (**Table 3**). The results of Cox regression analysis were visualized using forest plots (**Figures 2A, B**). These results suggest that elevated expression of MCP-4 in ovarian cancer tissues is associated with poor prognosis and that high expression of MCP-4 may serve as an independent risk factor for predicting the prognosis of patients with ovarian cancer.

**Table 3:** Cox regression analysis of overall survival of ovarian epithelial malignant tumors.

Variables	Univariate analysis			Multivariate analysis		
	HR	95% CI of HR	P-value	HR	95% CI of HR	*P*-value
MCP-4 expression (low vs high)	3.740	1.466-9.541	0.006	3.621	1.346-9.742	0.011
Age (<60 vs ≥60 years)	1.605	0.896-2.876	0.112			
FIGO stage (I-II vs III-IV)	7.788	3.250-18.666	0.000	5.589	2.111-14.802	0.001
Differentiation (well–moderate vs poor)	1.148	0.649-2.032	0.635			
Lymph node metastasis (no vs yes)	3.093	1.672-5.721	0.000	1.650	0.844-3.227	0.144

### MCP-4 promotes invasion, migration, and EMT of ovarian cancer cells

The expression of MCP-4 in normal ovarian epithelial cells (HOSEpiC) and two ovarian cancer cell lines (Caov3, ES-2) was examined. The results indicated that the expression of MCP-4 in the Caov3 and ES-2 cell lines was higher than that in HOSEpiC cells (**Figure 1C**).

**Figure 3:**
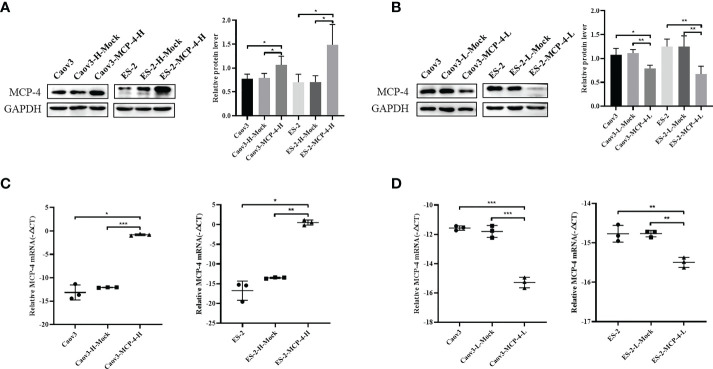
The protein and mRNA expression of MCP-4 in the overexpression/inhibition groups. **(A, B)** Representative images and quantitation of the western blotting showed that the protein expression of MCP-4 in the overexpression/knockdown groups (n = 3). GAPDH was used as an internal control. (**C**, **D**) The relative MCP-4 mRNA expression in the overexpression/inhibition groups (n = 3). Data are presented as mean ± SD. *P < 0.05; **P < 0.01; ***P < 0.001.

Overexpression of Caov3 and ES-2 cell lines of MCP-4 were constructed by lentiviral transfection, and inhibition cell lines of MCP-4 were constructed by siRNA transfection. RT-PCR and western blotting confirmed increased/decreased mRNA and protein levels of MCP-4 in overexpression/inhibition cell lines (**Figures 3A–D**).

**Figure 4:**
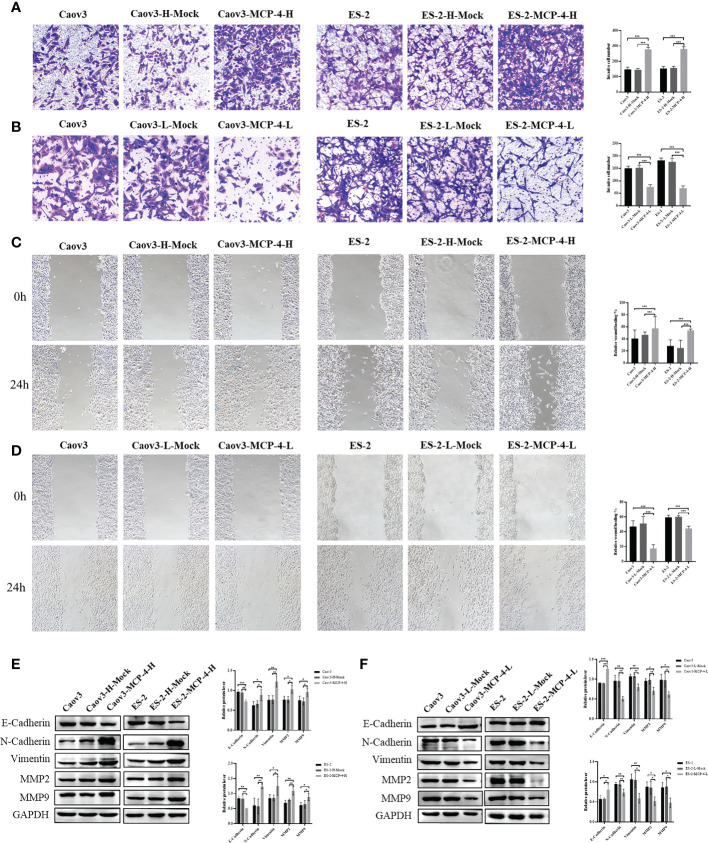
MCP-4 promoted invasion, migration, and EMT in ovarian cancer cells. **(A)** Overexpression of MCP-4 promoted invasion of Caov3 and ES-2 cell lines (n = 9; ×200) **(B)** Inhibition of MCP-4 expression suppressed ovarian cancer cells invasion (n = 9; ×200). **(C)** Overexpression of MCP-4 promoted migration of Caov3 and ES-2 cell lines (n = 9; ×100) **(D)** Inhibition of MCP-4 expression suppressed ovarian cancer cells migration (n = 9; ×100). **(E, F)** Representative images and quantitation of the western blotting showed that the protein expression of E-cadherin, N-cadherin, vimentin, MMP2, and MMP9 in the MCP-4 overexpression/inhibition groups (n = 3). GAPDH was used as an internal control. Data are presented as mean ± SD. *P < 0.05; **P < 0.01; ***P < 0.001.

To investigate the effect of MCP-4 on the migration and invasion abilities of ovarian cancer cells, we performed invasion and wound healing tests. The results showed that the overexpression of MCP-4 significantly enhanced the invasive ability of Caov3 and ES-2 cells (P < 0.05) (**Figure 4A**), whereas the inhibition of MCP-4 expression decreased the invasive ability of ovarian cancer cells (*P* < 0.05) (**Figure 4B**). After MCP-4 overexpression, the wound healing speed of Caov3 and ES-2 cells was significantly higher than that of the control group (*P* < 0.05) (**Figure 4C**). In addition, when MCP-4 was overexpressed, the levels of N-cadherin, vimentin, MMP-2, and MMP-9 also increased, and the level of E-cadherin decreased (**Figure 4E**). Overexpression of MCP-4 promotes epithelial mesenchymal transition in ovarian cancer. An opposite trend was observed when MCP-4 expression was inhibited (**Figures 4D, F**). These results confirm that MCP-4 can promote the invasion, migration, and EMT of ovarian cancer cells.

### MCP-4 inhibits apoptosis of ovarian cancer cells

**Figure 5:**
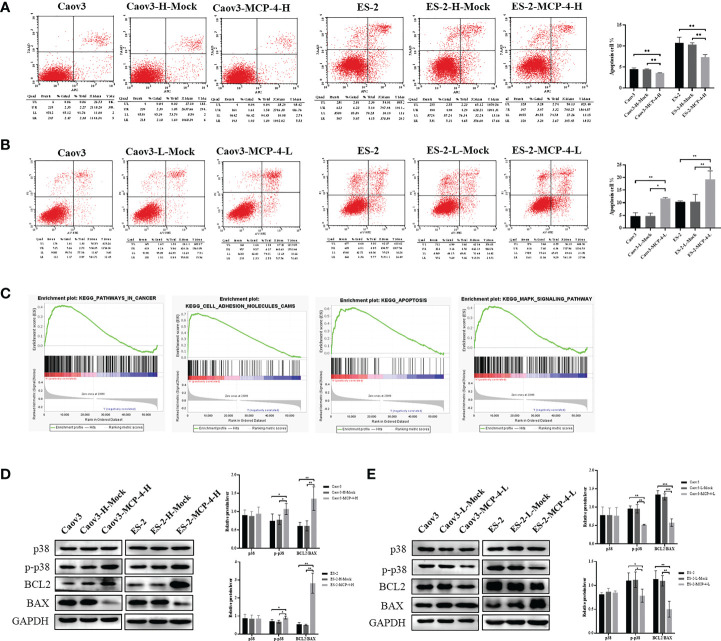
MCP-4 decreased cell apoptosis and activated the p38 MAPK signaling pathway. **(A)** Overexpression of MCP-4 decreased cell apoptosis of Caov3 and ES-2 cell lines **(B)** Inhibition of MCP-4 expression increased ovarian cancer cells apoptosis. **(C)** GSEA analysis of MCP-4 related enrichment gene sets. KEGG_PATHWAYS_IN_CANCER (NES = 1.64, P-value = 0.024, FDR = 0.059); KEGG_APOPTOSIS (NES = 2.20, P-value = 0.000, FDR = 0.000); KEGG_CELL_ADHESION_MOLECULES_CAMS (NES = 2.39, P-value = 0.000, FDR = 0.000); KEGG_MAPK_SIGNALING_PATHWAY (NES = 1.69, P-value = 0.012, FDR = 0.046) **(D, E)** Representative images and quantitation of the western blotting showed that the protein expression of p38 MAPK, p-p38 MAPK, BCL2, and BAX in the MCP-4 overexpression/inhibition groups (n = 3). GAPDH was used as an internal control. Data are presented as mean ± SD. *P < 0.05; **P < 0.01; ***P < 0.001.

Flow cytometry results showed that compared with the control group, the overall apoptosis rate of Caov3 and ES-2 cells was significantly reduced after MCP-4 overexpression (*P* < 0.05) (**Figure 5A**), and when MCP-4 expression was inhibited, the apoptosis rate was significantly increased (*P* < 0.05) (**Figure 5B**). Western blotting showed that the expression of anti-apoptotic protein BCL2 was upregulated, the expression of pro-apoptotic protein BAX was downregulated in the MCP-4 overexpression group, and the ratio of BCL2/BAX was significantly increased (*P* < 0.05) (**Figure 5D**). The results of MCP-4 inhibited expression group showed contradictory results (*P* < 0.05) (**Figure 5E**). These results indicate that MCP-4 could inhibit the apoptosis of ovarian cancer cells.

### MCP-4 activates p38 MAPK signaling pathways

The ovarian serous cystadenocarcinoma dataset downloaded from TCGA database was divided into the MCP-4 high-expression group and MCP-4 low-expression group according to the median, followed by GSEA enrichment analysis. These results suggest that MCP-4 is associated with pathways in cancer, cell adhesion molecules, apoptosis, and MAPK signaling pathway, which is consistent with the above experimental results that MCP-4 promotes migration and invasion and inhibits apoptosis in ovarian cancer cell lines (**Figure 5C**). Western blotting confirmed that MCP-4 activated the p38 MAPK signaling pathway. Overexpression of MCP-4 increased the expression of phosphorylated p38 MAPK (Thr180/Tyr182) (*P* < 0.05) (**Figure 5D**), whereas inhibition of MCP-4 decreased the expression of phosphorylated p38 MAPK (*P* < 0.05) (**Figure 5E**). However, the expression level of total p38 MAPK remained unchanged.

### MCP-4 promotes metastasis in a nude mouse peritoneal xenograft tumor model

**Figure 6:**
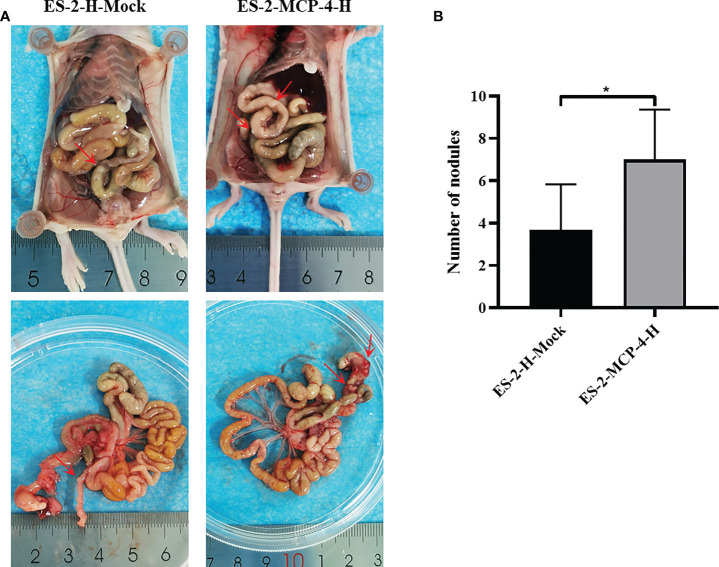
MCP-4 promotes metastasis in a nude mouse peritoneal xenograft tumor model. **(A)** After being sacrificed, dissemination of tumor in nude mice was assessed, and the metastasis nodules were marked by red arrows. **(B)** Average number of the peritoneal tumor nodules of each group were quantified (n = 6). Data are presented as mean ± SD. *P < 0.05.

MCP-4 overexpressing and control cells were intraperitoneally injected into nude mice. On the 25th day after intraperitoneal inoculation, nude mice were sacrificed and dissected. In order to objectively evaluate the metastasis ability regulated by MCP-4, all the metastatic nodules were collected, and the results showed that the number of metastatic nodules in the MCP-4-overexpression group were significantly higher than those in the control group (**Figures 6A, B**). These data indicates that MCP-4 significantly promotes tumor metastasis, and plays an important role in the malignant process of ovarian cancer.

## Discussion

Ovarian cancer is a common gynecological malignancy in women, and its incidence is second only to that of cervical and endometrial cancers. Intraperitoneal spread, extraperitoneal spread, and chemotherapy resistance are the main reasons for the low survival rate of ovarian cancer patients (1). The overall five-year survival rate of patients with advanced epithelial ovarian cancer (EOC) is approximately 30%. However, the overall five-year survival rate for patients diagnosed earlier is greater than 70% (11). Therefore, there is an urgent issue to explore rapid ovarian cancer biomarkers with high sensitivity and specificity.

Chemokines are the largest subfamily of cytokines and can be further subdivided into four major groups based on the position of the first two cysteine residues in their amino acid sequences: CC-chemokines, CXC-chemokines, C-chemokines, and CX3C-chemokines (12). Chemokines play important roles in inflammation, infection, internal environmental homeostasis, and tumor development and progression (13). MCP-4 is a member of the chemokine CC family. It has an α-helix at the carboxyl-terminus, two disulfide bonds near the amino-terminus, and three antiparallel β-sheets in the middle. It has a structure like other CC-chemokines and has high homology with MCP-1, MCP-2, and MCP-3 (14). Studies have found that the expression of MCP-4 is elevated in various diseases, such as asthma (15), nephritis (16), atherosclerosis (17), atopic dermatitis (18), and rheumatoid arthritis (19). Furthermore, it is involved in the immune response by recruiting inflammatory cells at the inflammation site. Recent studies have revealed that MCP-4 plays an important role in tumorigenesis.

OY et al. (7) reported that the expression of MCP-4 was elevated in colorectal cancer serum and correlated with poor prognosis, which could be used as a biomarker for distant metastasis and recurrence of colorectal cancer. The expression of MCP-4 is significantly higher in Helicobacter pylori-positive gastric cancer tissues than in normal gastric tissues (8). To date, no study has reported the expression and mechanism of MCP-4 in ovarian cancer. In this study, we confirmed that MCP-4 expression is elevated in ovarian cancer using IHC. Data from the TCGA and GEO databases further confirmed this result. The expression of MCP-4 was correlated with an advanced FIGO stage. Kaplan–Meier analysis showed that the overall survival of patients with high MCP-4 expression was significantly shorter. Cox analysis showed that MCP-4 was an independent risk factor affecting the prognosis of patients with ovarian cancer. In addition, western blotting confirmed that MCP-4 expression was higher in ovarian cancer cell lines than in normal ovarian epithelial cells. Therefore, MCP-4 may play an important role in ovarian cancer and be a predictor of ovarian cancer prognosis.

In our study, when MCP-4 was overexpressed in Caov3 and ES-2 cell lines, the expression of MMP-2 and MMP-9 were upregulated. EMT-related proteins, such as N-cadherin and vimentin, were increased, while E-cadherin was decreased. The opposite trend was observed when siRNA was used to inhibit the expression of MCP-4. In addition, we found that the apoptosis rate of ovarian cancer cells decreased when MCP-4 was overexpressed and increased when MCP-4 was inhibited. These results suggest that MCP-4 promotes malignant behavior in ovarian cancer cells. FB et al. (20) analyzed key immune-related proteins in fine-needle aspiration (FNA) biopsy samples from patients with breast cancer (n = 25) and benign lesions (n = 32). They found that MCP-4 was differentially expressed in breast cancer and benign lesions, and that there was a clear correlation with Ki67 expression. Thus, MCP-4 may promote the progression of breast cancer. AA et al. (21) analyzed the cytokine expression profiles of metastasis-derived colorectal cancer cell lines and cell lines derived from primary colorectal cancer and found that the expression level of MCP-4 was higher in metastatic colorectal cancer cell lines, suggesting that MCP-4 may be associated with colorectal cancer metastasis. OY et al. (7) examined the serum concentration of MCP-4 in colorectal cancer patients and showed that high concentrations of MCP-4 were significantly associated with old age, advanced T-stage, distant metastasis, and UICC stage. MCP-4 is an independent prognostic factor affecting disease-free survival and overall survival in colorectal cancer. These studies suggest that MCP-4 plays an important role in the development of various malignant tumors. Therefore, it is of great significance to investigate the expression and mechanism of MCP-4 in ovarian cancer.

To further explore the mechanisms by which MCP-4 affects the malignant biological behavior of ovarian cancer cells, we performed gene set enrichment analysis using GSEA software and found that MCP-4 is associated with cell adhesion molecules, apoptosis, cancer pathways, and MAPK signaling pathways. Traditional MAPKs include four major categories: ERK1/2, JNK, p38 MAPK, and ERK5 (22). They can be activated by a variety of growth factors or cytokines, thereby regulating a variety of cellular activities, including cell proliferation, differentiation, apoptosis, invasion, and hematopoiesis (23–25). Studies have confirmed that the p38 MAPK signaling pathway is often overactivated in malignant tumors and plays an important role in the malignant progression of ovarian cancer (26–28). ZJ et al. (29) found that galectin-1 enhanced ovarian cancer cell metastasis and EMT by promoting activation of the p38 MAPK/JNK signaling pathway. The combination of p38 MAPK inhibitors inhibits the growth and metastasis of olaparib-resistant ovarian cancer cells (30). p38 MAPK inhibitors combined with gemcitabine carboplatin improve progression-free survival (PFS) in patients with recurrent platinum-sensitive ovarian cancer (31). MCP-4 is a member of the CC-chemokine family and shares 67% sequence homology with MCP-1 (32). LJ et al. (33) showed that MCP-1 can upregulate the expression of p-MEK, p-ERK, p-p38 MAPK, and p-JNK and promote the invasion and migration of osteosarcoma cells by activating the c-Raf/MAPK/AP-1 signaling pathway. Previous studies have also confirmed that MCP-1 is associated with p38 MAPK phosphorylation in breast cancer cells (34). In ovarian cancer, MCP-1 secreted by tumor-associated mesothelial cells activates the p38 MAPK signaling pathway and promotes migration and invasion of ovarian cancer cells (35). These studies suggest that MCP-1 plays an important role in the tumor microenvironment of ovarian cancer, and may be related to the activation of the p38 MAPK signaling pathway. The mechanism of action of MCP-1 in malignancy has been extensively investigated, but it is still unclear how MCP-4 mediates malignant progression of ovarian cancer. Therefore, we examined the changes in the p38 MAPK signaling pathway in ovarian cancer cell lines with MCP-4 overexpression or inhibition. The results showed that overexpression of MCP-4 increased the expression of phosphorylated p38 MAPK, whereas total p38 MAPK expression did not change. The inhibition of MCP-4 expression in ovarian cancer cells showed the opposite trend. We have previously shown that MCP-4 promotes invasion, migration and EMT in ovarian cancer cells. In conclusion, MCP-4 may contribute to the malignant biology of ovarian cancer by activating the p38 MAPK signaling pathway.

In malignant tumors, tumor cells are considered one of the main sources of chemokines, and cells in the tumor stroma produce chemokines in response to stimulation (36). Under the action of specific chemokines, different types of immune cells migrate into the tumor microenvironment and regulate tumor immunity. In addition, chemokines can target non-immune cells in the tumor microenvironment, such as tumor cells and vascular endothelial cells, to influence tumor cell proliferation, invasion, migration, angiogenesis, and other malignant biological behaviors (37). Chemokines also promote communication among tumor cells, immune cells, mesenchymal cells, and endothelial cells, which play an important role in the development and metastasis of malignant tumors (38, 39). Our study confirmed that MCP-4 is upregulated in ovarian cancer and can promote cell invasion, migration and tumor metastasis. However, whether stromal cells, such as cancer-associated fibroblasts and cancer-associated mesothelial cells, can also express MCP-4 in ovarian cancer and its effects in the progression of ovarian cancer needs to be further investigated.

## Conclusion

In conclusion, we demonstrated for the first time that MCP-4 is overexpressed in ovarian cancer and is associated with poor prognosis. MCP-4 promotes ovarian cancer cell invasion, migration, and EMT and inhibits apoptosis. These malignant biological behaviors mediated by MCP-4 are related to the activation of the p38MAPK signaling pathway. Comprehensively elucidating the function of MCP-4 and its mechanism of action in ovarian cancer is important for identifying molecular markers for early diagnosis, targeted therapy, and prognosis evaluation.

## Data availability statement

The original contributions presented in the study are included in the article/supplementary material. Further inquiries can be directed to the corresponding authors.

## Ethics statement

The studies involving human participants were reviewed and approved by Ethics Committee of Shengjing Hospital Affiliated to China Medical University. The patients/participants provided their written informed consent to participate in this study. The animal study was reviewed and approved by Ethics Committee of Shengjing Hospital Affiliated to China Medical University.

## Author contributions

SL, YH, XL, and BL conceived and designed the experiments. SL performed the experiments. OL and XL performed data mining and analysis. SL and XL contributed to writing the manuscript. XL helped modify the manuscript. All authors contributed to the article and approved the submitted version.

## Funding

This work was supported by National Natural Science Foundation of China (No. 82173130), Key R&D Guidance Plan Project in Liaoning Province (2019JH8/10300022), and Beijing Kanghua Foundation for the Development of Traditional Chinese and Western Medicine Gynecological Oncology Special Research Fund (KH-2021-LLZX-010).

## Conflict of interest

The authors declare that the research was conducted in the absence of any commercial or financial relationships that could be construed as a potential conflict of interest.

## Publisher’s note

All claims expressed in this article are solely those of the authors and do not necessarily represent those of their affiliated organizations, or those of the publisher, the editors and the reviewers. Any product that may be evaluated in this article, or claim that may be made by its manufacturer, is not guaranteed or endorsed by the publisher.
